# Supplementary material for: MIR390 Is Involved in Regulating Anthracnose Resistance in Apple
Source: Plants (Basel). 2022 Nov 29;11(23):3299. doi: 10.3390/plants11233299 (PMC9736487; doi:10.3390/plants11233299)
Supplement: Supplementary file 1 [file plants-11-03299-s001.zip › plants-2044227-supplementary.pdf]

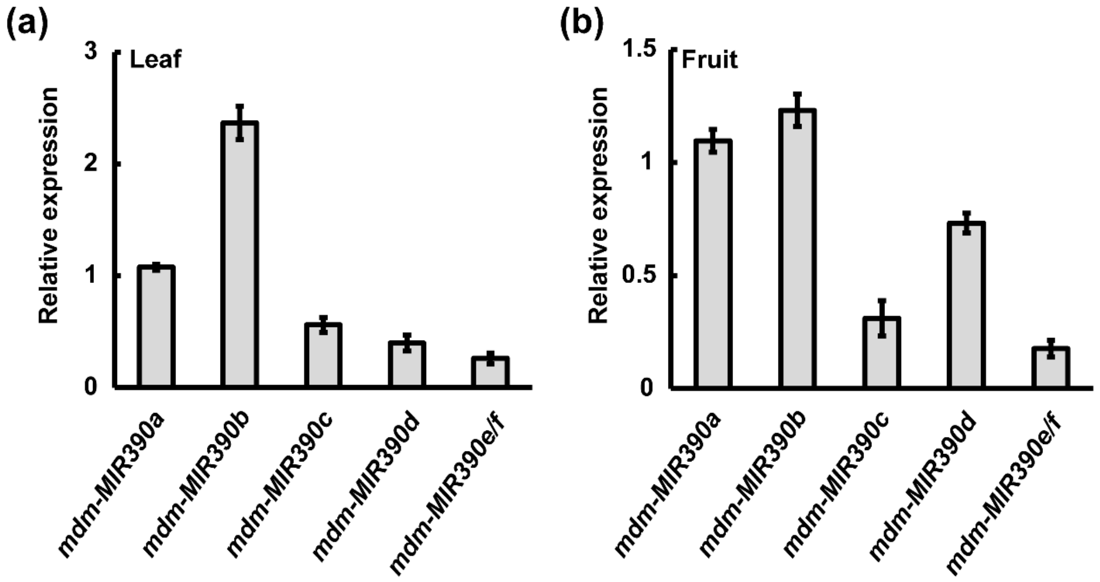

Supplementary Figure S1. Expression analysis of *mdm-MIR390a*, *mdm-MIR390b*, *mdm-MIR390c*, *mdm-MIR390d* and *mdm-MIR390e/f* in ‘Hanfu’ leaf and fruit. **(a)** qRT-PCR was used to examine *mdm-MIR390s* in apple leaf. The precursor sequences of *mdm-MIR390e* and *mdm-MIR390f* were the same, identical primes were used. The vertical bars represent SDs (n=3). **(b)** qRT-PCR was used to examine *mdm-MIR390s* in apple fruit. The precursor sequences of *mdm-MIR390e* and *mdm-MIR390f* were the same, identical primes were used. The vertical bars represent SDs (n=3).

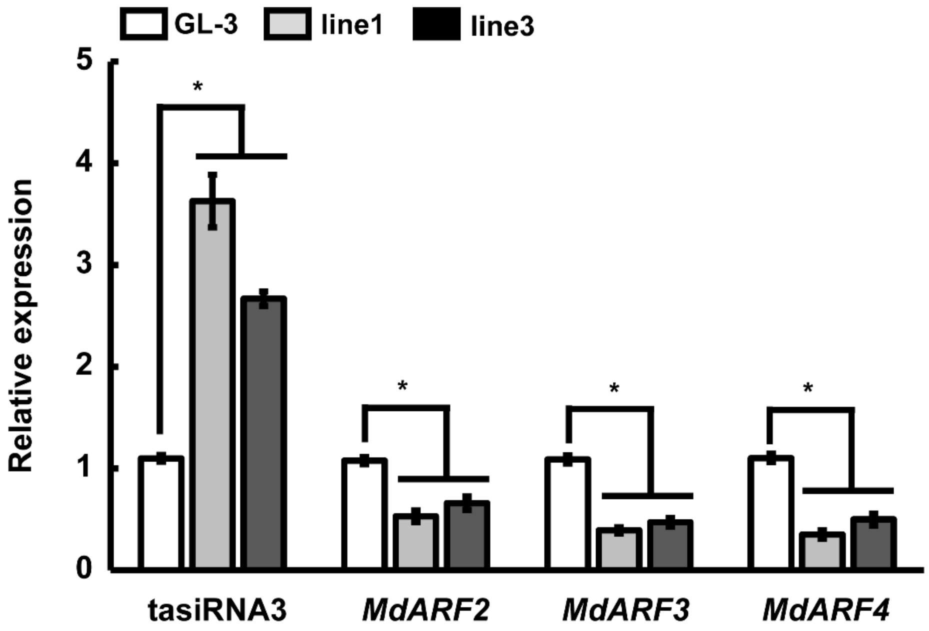

Supplementary Figure S2. Expression analysis of tasiRNA3, *MdARF2*, *MdARF3* and *MdARF4* in 'GL-3' and *MIR390b*-overexpressing plants. Leaves of 4-week-old 'GL-3', *MIR390b*-line1 and *MIR390b*-line3 plants were harvested. Expression of tasiRNA3*, MdARF2, MdARF3 and MdARF4* were measured in control and transgenic apple plants by qRT-PCR. The vertical bars represent SDs (n=3). '*' represents $P < 0.05$ (Student's *t*-test).

Supplementary Table S1. Primer sequences used in this study.

Primer	Sequence	Use
mdm-MIR390b-F	GTGTGGAAGAATCTGTTAAGCTCA	Precursor cloning
mdm-MIR390b-R	GTGTAAGAAGAAGCCATGAAACTCA	Precursor cloning
mdm-MIR390a-QF	AGTAAGGGAGAATCTGTAAAG	qRT-PCR
mdm-MIR390a-QR	AGTATGAAGAAGCCATGAAAC	qRT-PCR
mdm-MIR390b-QF	GTGTGGAAGAATCTGTTAAGCTCA	qRT-PCR
mdm-MIR390b-QR	GTGTAAGAAGAAGCCATGAAACTCA	qRT-PCR
mdm-MIR390c-QF	AGTAAGGGAGGATCTGTAAAG	qRT-PCR
mdm-MIR390c-QR	AGTAAGAAGAAGCCATGAAAC	qRT-PCR
mdm-MIR390d-QF	AGTAAGGGAGGATCTGTAAAG	qRT-PCR
mdm-MIR390d-QR	AGTAAGAAGAAGCCATGAAAC	qRT-PCR
mdm-MIR390e-QF	AGTGTGGAAGAATCTGTTAAG	qRT-PCR
mdm-MIR390e-QR	AGTAAGAAGAAGCCATGAAGC	qRT-PCR
mdm-MIR390f-QR	AGTGTGGAAGAATCTGTTAAG	qRT-PCR
mdm-MIR390f-QR	AGTAAGAAGAAGCCATGAAGC	qRT-PCR
MdPR2-QF	CATTCGTCTAGATTATGCTCTTTCCACAG	qRT-PCR
MdPR2-QR	TGTTGTTGCCGTCCCACCAGC	qRT-PCR
MdPR3-1-QF	CTTTCGTTGCTGCTGCTCGGTC	qRT-PCR
MdPR3-1-QR	TCTGGTGCACTTTCCCATCCTCC	qRT-PCR
MdPR10-1-QF	CACCTCCGTCATCCCCCCTGC	qRT-PCR
MdPR10-1-QR	CCTTGTCAACCCCATCAATTCTGTG	qRT-PCR
MdPR10-2-QF	GCAAACTACTCATACGCCTACAC	qRT-PCR
MdPR10-2-QR	GCTCTTCCTTGATCTCAACATC	qRT-PCR
MdARF2-QF	ATCCACTTCCAATGCCCAGG	qRT-PCR
MdARF2-QR	AAACTGGCTCTCAAGGTCGG	qRT-PCR
MdARF3-QF	CTTATGATCTCCCGCCCCAC	qRT-PCR

**Primer**	**Sequence**	**Use**
MdARF3-QR	CATTGCCTCAACGTCCTCCT	qRT-PCR
MdARF4-QF	CCCACCTTTGAGCATCCAGT	qRT-PCR
MdARF4-QR	TTGCGGGCGGTTTACAGTAT	qRT-PCR
MdEF-1α-F	ATTCAAGTATGCCTGGGTGC	qRT-PCR
MdEF-1α-R	CAGTCAGCCTGTGATGTTCC	qRT-PCR
miR390-RT	CTCAACTGGTGTCGTGGAGTCCGGCAAT TCAGTTGAGGGCGCTAT	qRT-PCR
miR390-QF	ACACTCCAGCTGGGAAGCTCAGGA	qRT-PCR
miR390-QR	AACTGGTGTCGTGGAG	qRT-PCR
miR390probe	FAM-TTCAGTTGAGGGCGCTAT-TAMRA	qRT-PCR
tasiRNA3-RT	CTCAACTGGTGTCGTGGAGTCCGGCAAT TCAGTTGAGGAGATCTT	qRT-PCR
tasiRNA3-QF	ACACTCCAGCTGGGTTCTTGACCT	qRT-PCR
tasiRNA3-QR	AACTGGTGTCGTGGAG	qRT-PCR
tasiRNA3probe	FAM-TTCAGTTGAGGAGATCTT-TAMRA	qRT-PCR
5SrRNA-RT	GTCACATCGTATCGTGAAGCTGCGCAGC TGATGTGACTGGATTGG	qRT-PCR
5SrRNA-QF	TGCACTAGCGTGTAGAGGAACC	qRT-PCR
5SrRNA-QR	ACATCGTATCGTGAAG	qRT-PCR
5SrRNAprobe	FAM-CTGATGTGACTGGATTGG-TAMRA	qRT-PCR
